# Oxacillinase-181 Carbapenemase-Producing *Klebsiella pneumoniae* in Neonatal Intensive Care Unit, Ghana, 2017–2019

**DOI:** 10.3201/eid2609.200562

**Published:** 2020-09

**Authors:** Appiah-Korang Labi, Karen L. Nielsen, Rasmus L. Marvig, Stephanie Bjerrum, Christabel Enweronu-Laryea, Marc Bennedbæk, Mercy J. Newman, Prosper K. Ayibor, Leif P. Andersen, Jørgen A.L. Kurtzhals

**Affiliations:** Korle-Bu Teaching Hospital, Accra, Ghana (A.-K. Labi);; Copenhagen University Hospital, Rigshospitalet, Copenhagen, Denmark (A.-K. Labi, K.L. Nielsen, R.L. Marvig, M. Bennedbæk, L.P. Andersen, J.A.L. Kurtzhals);; University of Copenhagen, Copenhagen (A.-K. Labi, S. Bjerrum, J.A.L. Kurtzhals);; University of Ghana Medical School, Accra (C. Enweronu-Laryea, M.J. Newman);; 37 Military Hospital, Accra (P.K. Ayibor)

**Keywords:** neonatal intensive care unit, Ghana, Klebsiella pneumoniae, whole-genome sequencing, bloodstream infection, carbapenemase-producing, bacteria, carbapenem, carbapenemase, antimicrobial resistance

## Abstract

We sequenced 29 carbapenemase-producing *Klebsiella pneumoniae* isolates from a neonatal intensive care unit in Ghana. Twenty-eight isolates were sequence type 17 with *bla*_OXA-181_ and differed by 0–32 single-nucleotide polymorphisms. Improved surveillance and infection control are needed to characterize and curb the spread of multidrug-resistant organisms in sub-Saharan Africa.

Carbapenems are antimicrobial drugs of last resort for infections caused by multidrug-resistant gram-negative bacteria. Therefore, the global spread of carbapenemase-producing *Enterobacteriaceae*, which are resistant to carbapenems, is troubling (*1*,*2*). Because of the high number of deaths associated with infections caused by these bacteria, the World Health Organization classifies *Enterobacteriaceae* as priority organisms for which new antimicrobial drugs are urgently needed (*3*).

Oxacillinase (OXA)-48–like carbapenemases are among the most common carbapenemases in Enterobacterales; of the OXA-48–like enzymes, OXA-181 is the second most common type (*2*). OXA-48 *Klebsiella pneumoniae* is considered endemic to North Africa and the Middle East; OXA-181 *Klebsiella pneumoniae* is endemic to the Indian subcontinent. However, nosocomial outbreaks of OXA-181 have occurred in sub-Saharan Africa (*2*). We describe the epidemiology and clonal spread of OXA-181–producing *Klebsiella pneumoniae* in a neonatal intensive care unit (NICU) in Ghana. The Institutional Review Board of the Korle-Bu Teaching Hospital granted ethics approval (no. IRB/0025/2017) for this study.

## The Study

We whole-genome sequenced 29 carbapenemase-producing *K. pneumoniae* isolates: 18 from neonatal carriage (isolates from swabs of neonates) (*4*), 3 from the NICU environment (cots and trolley handles, incubator doors, tables), and 8 from neonatal bloodstream infections. These samples were isolated from the NICU of Korle-Bu Teaching Hospital (Accra, Ghana) from September 2017 through February 2019 (*5*) (Table; Appendix).

**Table:** Characteristics of neonates and carbapenemase-producing *Klebsiella pneumoniae* isolates from Korle-Bu Teaching Hospital, Ghana, 2017–2019*

Patient	Sample	Sex	MOD	DOS	Birthweight, kg	Cubicle	Shared space	Date of isolation	ST	Capsular serotype	Prior AB use	AB use after BSI	DOA, d	Vital status
KP007	Carriage	F	CS	13	1.4	I	No	2017 Sep 21	17	KL25	AMK, CXC	NA	NA	Alive
KP010	Carriage	F	CS	21	1.1	II	No	2017 Sep 21	17	KL25	AMK, CXC	NA	NA	Alive
KP011	Carriage	M	SVD	18	0.9	I	No	2017 Sep 21	17	KL25	AMK, CXC	NA	NA	Dead
KP020	Carriage	F	CS	43	0.8	II	No	2017 Sep 21	48	KL62	AMK, CXC, MEM, CIP, VA	NA	NA	Alive
KP025	Carriage	F	SVD	15	1.2	III	No	2017 Sep 21	17	KL25	AMK, CXC	NA	NA	Alive
KP034	Carriage	M	CS	34	1.4	III	Yes	2017 Sep 21	17	KL25	AMK, CXC, CIP, MEM	NA	NA	Alive
KP035	Carriage	F	CS	30	1.4	III	Yes	2017 Sep 21	17	KL25	No	NA	NA	Alive
KP036	Carriage	F	SVD	36	1.3	III	Yes	2017 Sep 21	17	KL25	No	NA	NA	Alive
KP037	Carriage	M	SVD	12	2.1	III	Yes	2017 Sep 21	17	KL25	AMK, CXC	NA	NA	Alive
KP045	Carriage	F	SVD	11	1.7	III	No	2017 Sep 21	17	KL25	AMK, CXC	NA	NA	NA
KP047	Carriage	F	SVD	4	3.9	III	No	2017 Sep 21	17	KL25	AMK, CXC	NA	NA	Alive
KP052	Carriage	M	CS	12	3	III	Yes	2017 Sep 21	17	KL25	AMK, CXC	NA	NA	Alive
KP055	Carriage	M	CS	24	1.5	III	Yes	2017 Sep 21	17	KL25	AMK, CXC	NA	NA	Alive
KP056	Carriage	M	SVD	17	3.2	III	No	2017 Sep 21	17	KL25	AMK, CXC	NA	NA	Alive
K058	Carriage	M	SVD	33	1	I	No	2017 Sep 21	17	KL25	AMK, CXC	NA	NA	Alive
K221	Carriage	F	SVD	2	1.6	III	No	2017 Sep 21	17	KL25	AMK, CXC	NA	NA	Alive
KP233	Carriage	M	SVD	12	3.2	III	No	2018 Jan 19	17	KL25	AMK, CXC	NA	NA	Alive
KP242	Carriage	M	SVD	2	3.7	III	No	2018 Jan 19	17	KL25	AMK, CXC	NA	NA	Alive
KP0033	Blood	M	SVD	20	2.4	I	NA	2017 Oct 5	17	KL25	AMK, CXC, CAZ	MEM, CIP	10	Dead
KP0455	Blood	M	SVD	10	3.6	II	NA	2018 Jan 5	17	KL25	AMK, CXC	NA	12	Dead
KP0457	Blood	F	SVD	9	1.3	II	NA	2018 Jan 5	17	KL25	AMK, CXC	CIP	69	Alive
KP0879	Blood	M	SVD	13	4.4	III	NA	2018 Mar 19	17	KL25	MEM	MEM	NA	NA
KP2326	Blood	F	A	7	1.1	I	NA	2018 Dec 29	17	KL25	AMK, CXC	NA	9	Dead
KP2201	Blood	M	CS	10	1.3	I	NA	2018 Nov 24	17	KL25	AMK, CXC	MEM, CIP	42	Alive
KP2557	Blood	M	SVD	18	2.5	I	NA	2019 Feb 6	17	KL25	AMK, CXC	MEM	NA	Alive
KP2455	Blood	F	CS	10	NA	III	NA	2019 Jan 20	17	KL25	AMK, CXC	CIP	27	Alive
KP026	Cot					II		2017 Sep 21	17	KL25				
KP040	Cot					III		2017 Sep 21	17	KL25				
KP090	Trolley handle					III		2017 Sep 21	17	KL25				

*Blank spaces indicate not applicable. AB, antimicrobial drug; AMK, amikacin; BSI, bloodstream infections; CAZ, ceftazidime; CIP, ciprofloxacin; CS, cesarean section; CXC, cloxacillin; DOA, duration of admission; DOS, duration of stay before specimen collection; MOD, mode of delivery; MEM, meropenem; NA, not available; ST, multilocus sequence type.

**Figure 1:**
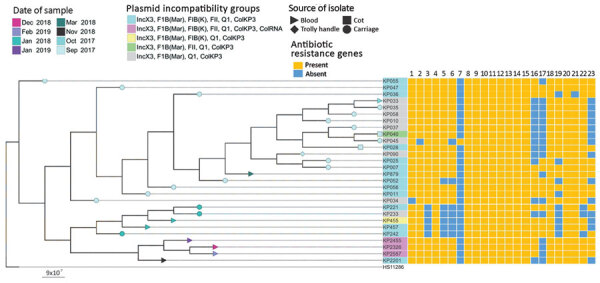
Phylogenetic tree of 28 carbapenemase-producing *Klebsiella pneumoniae* isolates and their acquired resistance genes from the neonatal intensive care unit at Korle-Bu Teaching Hospital, Accra, Ghana, 2017–2019. The tree was produced by analysis of single-nucleotide polymorphisms (SNPs) of core genomes. Maximum genetic distance was between isolates KP2201 and KP026, which differed by 32 SNPs. Tree used genome of *K. pneumoniae* reference strain HS11286 as outgroup. Lane 1, *rmt*B; lane 2, *aph(3")-lb*; lane 3, *aph(3')-la*; lane 4, *aph(6)-ld*; lane 5, *aac(3)-lld*; lane 6, *aadA2*; lane 7, *aadA2b*; lane 8, *bla*_OXA-181_; lane 9, *bla*_TEM-1B_; lane 10, *bla*_SHV-94_; lane 11, *bla*_CTX-M-15_; lane 12, *qnrS*; lane 13, *oqxA*; lane 14, *oqxB*; lane 15, *fosA*; lane 16, *mph(A)*; lane 17, *catA2*; lane 18, *sul2*; lane 19, *sul1*; lane 20, *tetA*; lane 21, *tetG*; lane 22, *dfrA12*; lane 23, *drfA14*. Scale bar indicates substitutions per site.

Twenty-eight of the 29 isolates were sequence type (ST) 17 and capsular type KL25. We excluded 1 isolate from further analysis that was ST48 and KL64. Core-genome phylogeny showed a close genetic relationship of all ST17 isolates (0–32 single-nucleotide polymorphism [SNP] differences; median 5 SNP differences), suggesting a localized outbreak (Figure 1). We estimated that the most recent common ancestor of the outbreak emerged in April 2017 (year 2017.3; 95% highest posterior density interval 2017.0–2017.6) with an estimated mean substitution rate of 2.1 × 10^–6^ SNPs/site/year (9.9 SNPs/year) (Appendix Figure).

All isolates were resistant to amoxicillin/clavulanic acid, gentamicin, amikacin, cefuroxime, ceftriaxone, ceftazidime, tazobactam/piperacillin, and ciprofloxacin. The isolates were susceptible to colistin and had MICs of <1 μg/mL. All outbreak isolates harbored the carbapenemase *bla*_OXA-181_ and extended-spectrum β-lactamase *bla*_CTX-M-15_ in addition to other β-lactamases (*bla*_TEM-1B_, *bla*_SHV-94_). We also found several genes encoding resistance to other antimicrobial drugs: aminoglycosides (*rmt*B, *aph(3¢')-Ib*, *aph(3¢)-Ia*, *aph(**6**)-Id*, *aac(**3**)-IId*, *aadA2*, *aadA2b*); fluoroquinolones (*qnrS*, *oqxA*, *oqxB*); fosfomycin (*fosA*); macrolide (*mph (A)*); phenicols (*catA2*); sulphonamides (*sul2*, *sul1*); tetracyclines (*tetA*, *tetG*); and trimethoprim (*dfrA1*2, *dfrA1*4) (Figure 1).

**Figure 2:**
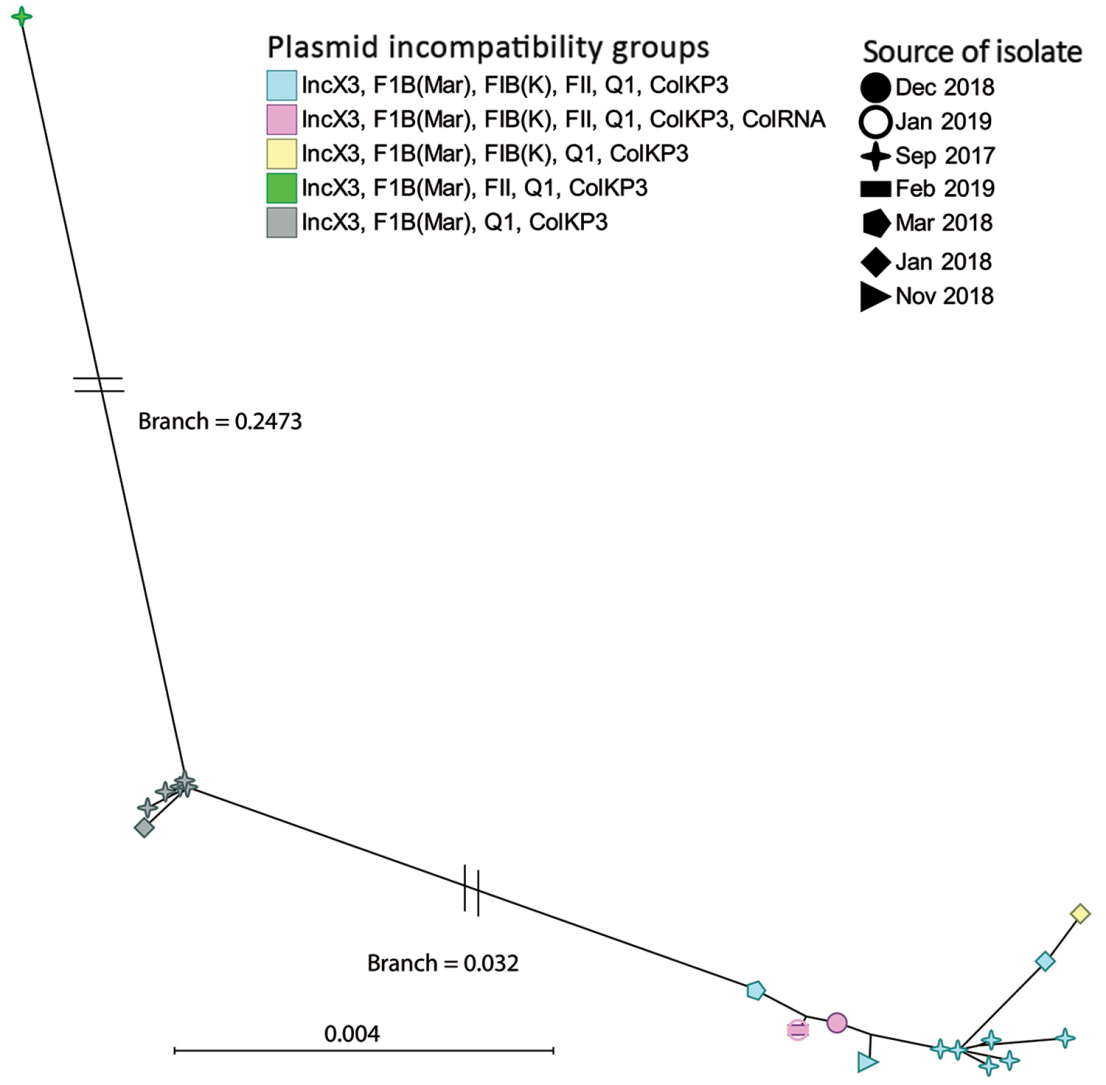
Binary rational tree illustrating genetic diversity (presence–absence of genes) of the accessory genome of carbapenemase-producing *Klebsiella pneumoniae* isolates from the neonatal intensive care unit at Korle-Bu Teaching Hospital, Accra, Ghana, 2017–2019. Different shapes represent different dates of organism isolation. Blue and green shapes evolved from the gray; pink and yellow evolved from the blue. Scale bar indicates genetic differences per site.

All isolates contained 4 common plasmid incompatibility (Inc) groups (IncX3, IncF1B (Mar), IncQ1, IncColKP3). Eighteen isolates also contained incompatibility groups IncFIB (K) and IncFII, and 3 contained additional IncColRNA (Figures 1, 2). Further analysis revealed that recently recovered isolates had more plasmid Inc groups than did older isolates (Figure 1). The accessory genome of the isolates showed large variation in gene content (Figure 2). These data illustrate that this variation existed at the time of the first sampling in September 2017, when the isolates formed 3 distinct clusters (Figure 2). The clustering is associated with differences in plasmid content of the isolates and represents the uptake or loss of 205 genes. On the basis of the phylogeny and metadata, we hypothesize that 4 major evolutionary events caused changes in Inc groups and the ancestor of the cluster of isolates with Inc groups IncX3, IncFIB, IncQ1 and ColKP3 (Figure 2).

A study in South Africa identified a fully closed plasmid carrying *bla*_OXA-181_ (*6*). Using the short-read sequencing applied in this study, we cannot determine whether *bla*_OXA-181_ is carried on a plasmid or located in the chromosome. Mapping of raw reads toward the fully closed plasmid reveals complete coverage across the whole plasmid for 24 of the 28 isolates; the remaining 4 most recent isolates had reads covering the whole plasmid (except for 4 genes). This finding might indicate these isolates have a similar plasmid containing *bla*_OXA-181_, although we cannot rule out that these reads might belong to other related plasmids and not the previously reported plasmid (*6*).

## Conclusions

We identified an outbreak of ST17 *K. pneumoniae* carrying *bla*_OXA-181_ in a NICU in Ghana. Outbreak isolates were resistant to all antimicrobial drugs commonly used to treat neonatal infections (although it was susceptible to colistin). Similar outbreaks of ST17 OXA-181–producing *K. pneumoniae* have been documented in South Africa (*7*), further confirming the spread of this type of resistance into nonendemic regions (*2*). Time-based phylogenetic analysis showed the outbreak isolates share a recent ancestor (approximately April 2017). This finding suggests that the outbreak strain had been introduced recently into the NICU or that the outbreak strain had limited genetic diversity because of a recent bottleneck or selective sweep in the outbreak strain population.

*K. pneumoniae* is an entry point of antimicrobial resistance into the family *Enterobacteriaceae* (*8*). Thus, carbapenemase-producing *K. pneumoniae* in the NICU might transmit resistance to other *Enterobacteriaceae* species. Other studies have associated *bla*_OXA-181_ with the insertion sequence element ISEcp1, which can spread cephalosporinases and extended-spectrum β-lactamases (*9*). In our study, all isolates possessed the IncX3 plasmid. This plasmid is self-transmissible and associated with worldwide dissemination of New Delhi metallo-β-lactamases 1 and 5 (*10*,*11*). Recent studies from countries in Africa have found *bla*_OXA-181_ carried on the IncX3 plasmid in *Enterobacteriaceae* species, including *K. pneumoniae* (*2*,*6*,*7*).

In Europe, the spread of carbapenem-resistant *K. pneumoniae* has been driven by 4 carbapenemase-positive clonal lineages that are often transmitted in hospitals (*8*). The isolates from the NICU were genetically diverse, especially in the plasmid content of the accessory genome. This diversity indicates the genome evolved rapidly, similar to isolates from an outbreak of *K. pneumoniae* in Beijing, China. In the outbreak in China, the isolates underwent rapid genotypic evolution mainly through rearrangement (including the gain and loss of genes) in the accessory genome (*12*). Antimicrobial pressure in hospitals might lead to adaptation and resistance transmission of *K. pneumoniae* in the hospital environment (*8*).

From our data, we infer the background transmission of carbapenemase-producing *K. pneumoniae* in the NICU before its detection. Neonatal carriage or environmental contamination by carbapenemase-producing *K. pneumoniae* might have started or maintained the outbreak. Improved surveillance of multidrug-resistant organisms, buttressed with improved infection prevention and control activities, are required to detect and control outbreaks in low-resource settings.

Appendix Additional information about methods of sampling *Klebsiella pneumoniae* isolates and analyzing their phylogenetic relationships.
