# Supplementary material for: Oxacillinase-181 Carbapenemase-Producing Klebsiella pneumoniae in Neonatal Intensive Care Unit, Ghana, 2017–2019
Source: Emerg Infect Dis. 2020 Sep;26(9):2235–8. doi: 10.3201/eid2609.200562 (PMC7454046; doi:10.3201/eid2609.200562)
Supplement: Appendix — Additional information about methods of sampling Klebsiella pneumoniae isolates and analyzing their phylogenetic relationships. [file 20-0562-Techapp-s1.pdf]

Article DOI: https://doi.org/10.3201/eid2609.200562

Oxacillinase-181 Carbapenemase-Producing *Klebsiella pneumoniae* in Neonatal Intensive Care Unit, Ghana, 2017–2019

Appendix

Detailed Methods

Sampling Hospital

The Korle-Bu Teaching Hospital Neonatal Intensive Care Unit (NICU) is a 55-bed facility, divided into three main cubicles with a higher number of patients in cubicle III compared to the others (Cubicle I—unstable/critical care, Cubicle II—preterm/low birthweight, Cubicle III—stable/pre-discharge normal birthweight) and a five-bed kangaroo mother care ward, in which babies are kept warm by their mothers' skin as an alternative to incubators (*1*). Neonates typically transition through a minimum of two cubicles before discharge. Unstable neonates in cubicles II and III may be transferred back to cubicle I. The unit admits ≈2,600 neonates/year (*2*) and receives regular visits by the hospital infection control nurse but has no surveillance for healthcare-associated infections and no screening program for multidrug-resistant (MDR) organisms.

Study Design

Over a 19-month period from September 2017 to February 2019 we conducted an interventional study to evaluate the effects of the World Health Organization multimodal hand hygiene strategy on bloodstream infections and carriage of MDR gram-negative bacteria at two NICUs at two different hospitals (NCT03755635 clinicaltrials.gov) (*3*). Korle-Bu Teaching Hospital acted as the intervention site.

We conducted two cross-sectional surveys in the NICU in September 2017 and January 2018 to determine carriage of MDR gram-negative bacteria (*4*). Pooled swabs from the neonates'

axilla, groin and peri-anal region were cultured for gram-negative bacteria on MacConkey agar. To better understand the role of the environment in the spread of healthcare-associated infections at the NICU, we conducted three environmental screenings of the NICU in September 2017, October 2017, and January 2018. Areas screened included incubator doors, cots, trolley handles, door handles, weighing scales, tables, and desks.

Blood cultures were collected by using the BACTEC culture system (Becton Dickinson, Maryland, USA) throughout the study period for all neonates at risk for sepsis or with clinically suspected sepsis. Epidemiologic data were prospectively extracted from clinical notes, including date of sampling for bloodstream infection.

Following the detection of $bla_{\text{OXA-181}}$ carbapenem-producing *K. pneumoniae* carriage at the NICU, we initiated an outbreak investigation using whole-genome sequencing (WGS) to understand transmission in the NICU. In total, 161 *K. pneumoniae* isolates were identified from carriage (n = 99), environment (n = 14) and blood cultures (n = 48). All 29 *K. pneumoniae* isolates with phenotypic carbapenem resistance were included in the sequencing study.

Infection Control Interventions

In response to the outbreak and the discovery of environmental contamination by MDR gram-negative bacteria, three major deep environmental cleaning exercises were conducted besides the routine cleaning at the NICU (October 2017, August 2018, March 2019). These were not part of the initial protocol but deemed necessary by the infection control unit. As part of the original protocol, an alcohol-based hand hygiene intervention using the WHO multimodal hand hygiene strategy (*3*,*5*) was instituted at the NICU to improve hand hygiene practice over a 6-month period (September 2018-March 2019).

Phenotypic Characterization of Isolates

All collected isolates were speciated using MALDI Biotyper (Bruker Daltonics®, Bremen, Germany). Antibiotic susceptibility testing was performed using the disc diffusion method and interpreted according to The European Committee on Antimicrobial Susceptibility Testing (EUCAST) guidelines (*6*). Antibiotic discs included amoxicillin-clavulanic acid, mecillinam, cefuroxime, ceftriaxone, ceftazidime, gentamicin, amikacin, ciprofloxacin, sulfamethoxazole-trimethoprim, meropenem and tigecycline from Oxoid (Basingstoke, UK). Susceptibility to colistin was tested using Micronaut Colistin MIC-Strip (Merlin Diagnostika

GmbH, Bornheim, Germany). Carbapenemase- and extended-spectrum beta-lactamases-(ESBL)-production were determined following EUCAST guidelines (*6*,*7*). These tests were performed using ROSCO (Taastrup, Denmark) phenotypic ESBL + AmpC beta-lactamase and Klebsiella pneumoniae carbapenemase + Metallo beta lactamase + oxacillinase (OXA)-48 carbapenemase Kit. Multidrug resistance was defined as non-susceptibility to ≥1 antibiotic in ≥3 antibiotic groups, with the following antibiotics used in the classification; gentamicin/amikacin, piperacillin tazobactam, meropenem, cefuroxime, cefotaxime, ciprofloxacin and amoxicillin-clavulanic acid (*8*).

Genome Sequencing and Analyses

We determined genetic relatedness of suspected outbreak isolates by WGS. DNA was extracted and purified with DNeasy Blood and Tissue kit (Qiagen, Hilden, Germany). Isolates were whole-genome sequenced on a MiSeq Instrument (Illumina, San Diego, CA, USA) using paired-end libraries (2× 250bp). Number of reads and total number of bases sequenced per isolate is listed in Appendix Table 1.

Genome assemblies and annotations were created using Bifrost pipeline (https://github.com/ssi-dk/bifrost), including SKESA assembly (*9*) and Prokka annotation (*10*). We analyzed the following in silico: resistance genes with ResFinder (*11*); multilocus sequence typing with MLST 2.0 (*12*); plasmid content using PlasmidFinder (*13*) and capsular types using Kaptive (*14*,*15*). Pan genome analysis was conducted by using GenAPI on default settings (Gabrielaite et al., unpub. data, https://www.biorxiv.org/content/10.1101/658476v1), and the differences in gene content were visualized in CLC genomics workbench where metadata was added. Manual BLAST (https://blast.ncbi.nlm.nih.gov/Blast.cgi) analysis of specific gene sequences was performed in Geneious Prime. We mapped raw reads from the isolates in this study toward plasmid CP034284.1 (GenBank accession no.) using Geneious Prime 2019.2.1.

We used BacDist to identify single nucleotide polymorphisms (SNPs) in each ST17 isolate relative to *Klebsiella pneumoniae* strain HS11286 reference genome (RefSeq assembly accession no. GCF_000240185.1) (BacDist: doi: 10.5281/zenodo.3667680) (https://github.com/MigleSur/BacDist). This reference sequence is from a published and fully closed genome of a *bla*$_{KPC-2}$ carbapenemase-producing *K. pneumoniae* (*16*). BacDist compared SNPs across all isolates to identify those that differed between the isolates, i.e., to identify SNPs

that have accumulated since the most recent common ancestor. BacDist filtered mutations to only retain SNPs at positions covered by at least 10 reads in all clones and to exclude mutations where all clones showed more than 80% non-reference reads at the given position. 4,725,103 of 5,333,943 nt in the reference genome was included in the analysis (i.e., covered by at least 10 reads in all isolates). Furthermore, BacDist used the identified SNPs to generate a maximum-likelihood phylogenetic tree with RAxML version 8.2.11 using a general time reversible model of nucleotide substitution (option -m GTRCAT). Pairwise genetic distances were measured by the number of SNPs reported by BacDist between any pair of isolates. Also, BacDist used ClonalFrame to identify and filter genomic regions with homologous recombination events, and no recombination events were identified.

Time-scaled phylogenetic reconstruction was performed using Bayesian Evolutionary Analysis Sampling Trees (BEAST) version 2.5.0 (*17*). BEAST analysis was run with HKY85 (Hasegawa, Kishino, and Yano 1985) DNA substitution model, lognormal relaxed clock, and exponential population growth. The choice of settings was based on our previous experience running BEAST (*18*–*22*), and on initial tests with the following models and priors: substitution models HKY and generalized times reversible clock models strict, relaxed exponential, and relaxed log normal; priors coalescent exponential population and coalescent constant population. A time-based phylogenetic tree was calculated from a chain of 300 million steps with sampling every 1,000 steps. The first 10% of steps were discarded as burn-in, and effective sample sizes and 95% highest posterior density (HPD) intervals (i.e., an interval in which the modeled parameter resides with 95% probability) were calculated by Tracer version 1.7.1 (*23*).

We explored the robustness of the BEAST analysis with both respect to use of outgroup (Appendix Table 2) and with respect to different models and priors (Appendix Table 3). First, we performed the time-based phylogenetic analysis with three variations with respect to prior definition of outgroup: (a) with no outgroup, (b) with KP055 (the isolate furthest from the other isolates in the maximum likelihood tree) as an outgroup, and (c) with reference strain HS11286 included as an outgroup. We obtained similar results from all three variations of the analysis, and all three analyses yielded effective sample sizes (ESS) of all parameters of ≥1,838 as calculated by Tracer (Appendix Table 2).

Second, we performed the time-based phylogenetic analysis with all 12 possible combinations of the initially tested models and priors (substitution models HKY and GTR; clock models strict, relaxed exponential, and relaxed log normal; priors coalescent exponential population and coalescent constant population). All 12 analyses yielded effective sample sizes (ESS) of all parameters of ≥2,762 and the time of most recent common ancestor (tMRCA) ranged from year 2017.05 to 2017.09 (Appendix Table 3).

Overall, we obtained similar results across the shown variations with either respect to outgroup (Appendix Table 2) or models and priors (Appendix Table 3), and all variations yielded ESS of all parameters that were adequate according to guidelines for running BEAST (https://beast.community). We have reported results with reference strain HS11286 as outgroup in the main manuscript; nonetheless, we find that all the shown results support the conclusion that the outbreak strains share a recent common ancestor from around early 2017.

Phylogenetic trees were visualized and annotated with metadata using CLC Genomics Workbench 12.0.3 (Qiagen). Raw reads of the *K. pneumoniae* genomes reported in this study are available in European Nucleotide Archive database under the accession no. PRJEB37523.

References

1. Conde-Agudelo A, Díaz-Rossello JL. Kangaroo mother care to reduce morbidity and mortality in low birthweight infants. Cochrane Database Syst Rev. 2014;4:CD002771. PubMed https://doi.org/10.1002/14651858.CD002771.pub3

2. Child Health Sub-BMC. In: Annual Report, Korle-Bu Teaching Hospital. Accra, Ghana: Korle-Bu Teaching Hospital. 2014. p. 68–85 [cited 2015 Feb 10]. https://kbth.gov.gh/web-forms-and-documents

3. Clinicaltrials.gov. Neonatal Sepsis at Neonatal Intensive Care Units in Ghana [cited 2019 Nov 22]. https://clinicaltrials.gov/ct2/show/NCT03755635

4. Labi A-K, Bjerrum S, Enweronu-Laryea CC, Ayibor PK, Nielsen KL, Marvig RL, et al. High carriage rates of multi-drug resistant gram-negative bacteria in neonatal intensive care units from Ghana. Open Forum Infect Dis. 2020;7:ofaa109.

5. Allegranzi B, Gayet-Ageron A, Damani N, Bengaly L, McLaws M-L, Moro M-L, et al. Global implementation of WHO's multimodal strategy for improvement of hand hygiene: a quasi-experimental study. Lancet Infect Dis. 2013;13:843–51. PubMed https://doi.org/10.1016/S1473-3099(13)70163-4

6. European Society of Clinical Microbiology and Infectious Disease. EUCAST guideline on detection of resistance mechanisms, version 2.0. 2017 [cited 2018 Apr 3]. http://www.eucast.org/resistance_mechanisms/

7. Haldorsen B, Giske CG, Hansen DS, Helgason KO, Kahlmeter G, Löhr IH, et al.; NordicAST CPE Study Group. Performance of the EUCAST disc diffusion method and two MIC methods in detection of *Enterobacteriaceae* with reduced susceptibility to meropenem: the NordicAST CPE study. J Antimicrob Chemother. 2018;73:2738–47. PubMed https://doi.org/10.1093/jac/dky276

8. Magiorakos A-P, Srinivasan A, Carey RB, Carmeli Y, Falagas ME, Giske CG, et al. Multidrug-resistant, extensively drug-resistant and pandrug-resistant bacteria: an international expert proposal for interim standard definitions for acquired resistance. Clin Microbiol Infect. 2012;18:268–81. PubMed https://doi.org/10.1111/j.1469-0691.2011.03570.x

9. Souvorov A, Agarwala R, Lipman DJ. SKESA: strategic k-mer extension for scrupulous assemblies. Genome Biol. 2018;19:153. PubMed https://doi.org/10.1186/s13059-018-1540-z

10. Seemann T. Prokka: rapid prokaryotic genome annotation. Bioinformatics. 2014;30:2068–9. PubMed https://doi.org/10.1093/bioinformatics/btu153

11. Zankari E, Hasman H, Cosentino S, Vestergaard M, Rasmussen S, Lund O, et al. Identification of acquired antimicrobial resistance genes. J Antimicrob Chemother. 2012;67:2640–4. PubMed https://doi.org/10.1093/jac/dks261

12. Larsen MV, Cosentino S, Rasmussen S, Friis C, Hasman H, Marvig RL, et al. Multilocus sequence typing of total-genome-sequenced bacteria. J Clin Microbiol. 2012;50:1355–61. PubMed https://doi.org/10.1128/JCM.06094-11

13. Carattoli A, Zankari E, García-Fernández A, Voldby Larsen M, Lund O, Villa L, et al. In silico detection and typing of plasmids using PlasmidFinder and plasmid multilocus sequence typing. Antimicrob Agents Chemother. 2014;58:3895–903. PubMed https://doi.org/10.1128/AAC.02412-14

14. Wick RR, Heinz E, Holt KE, Wyres KL. Kaptive Web: user-friendly capsule and lipopolysaccharide serotype prediction for *Klebsiella* genomes. J Clin Microbiol. 2018;56:e00197–18. PubMed https://doi.org/10.1128/JCM.00197-18

15. Wyres KL, Wick RR, Gorrie C, Jenney A, Follador R, Thomson NR, et al. Identification of *Klebsiella* capsule synthesis loci from whole genome data. Microb Genom. 2016;2:e000102. PubMed https://doi.org/10.1099/mgen.0.000102

16. Liu P, Li P, Jiang X, Bi D, Xie Y, Tai C, et al. Complete genome sequence of *Klebsiella pneumoniae* subsp. *pneumoniae* HS11286, a multidrug-resistant strain isolated from human sputum. J Bacteriol. 2012;194:1841–2. PubMed https://doi.org/10.1128/JB.00043-12

17. Bouckaert R, Vaughan TG, Barido-Sottani J, Duchêne S, Fourment M, Gavryushkina A, et al. BEAST 2.5: An advanced software platform for Bayesian evolutionary analysis. PLOS Comput Biol. 2019;15:e1006650. PubMed https://doi.org/10.1371/journal.pcbi.1006650

18. Feliziani S, Marvig RL, Luján AM, Moyano AJ, Di Rienzo JA, Krogh Johansen H, et al. Coexistence and within-host evolution of diversified lineages of hypermutable *Pseudomonas aeruginosa* in long-term cystic fibrosis infections. PLoS Genet. 2014;10:e1004651. PubMed https://doi.org/10.1371/journal.pgen.1004651

19. Knudsen GM, Nielsen JB, Marvig RL, Ng Y, Worning P, Westh H, et al. Genome-wide-analyses of *Listeria monocytogenes* from food-processing plants reveal clonal diversity and date the emergence of persisting sequence types. Environ Microbiol Rep. 2017;9:428–40. PubMed https://doi.org/10.1111/1758-2229.12552

20. Markussen T, Marvig RL, Gómez-Lozano M, Aanæs K, Burleigh AE, Høiby N, et al. Environmental heterogeneity drives within-host diversification and evolution of *Pseudomonas aeruginosa.* MBio. 2014;5:e01592–14. PubMed https://doi.org/10.1128/mBio.01592-14

21. Marvig RL, Dolce D, Sommer LM, Petersen B, Ciofu O, Campana S, et al. Within-host microevolution of *Pseudomonas aeruginosa* in Italian cystic fibrosis patients. BMC Microbiol. 2015;15:218. PubMed https://doi.org/10.1186/s12866-015-0563-9

22. Marvig RL, Johansen HK, Molin S, Jelsbak L. Genome analysis of a transmissible lineage of *pseudomonas aeruginosa* reveals pathoadaptive mutations and distinct evolutionary paths of hypermutators. PLoS Genet. 2013;9:e1003741. PubMed https://doi.org/10.1371/journal.pgen.1003741

23. Rambaut A, Drummond AJ, Xie D, Baele G, Suchard MA. Posterior summarization in Bayesian phylogenetics using Tracer 1.7. Syst Biol. 2018;67:901–4. PubMed https://doi.org/10.1093/sysbio/syy032

Appendix Table 1. Sequenced isolates of *Klebsiella pneumoniae* from a neonatal intensive care unit, Ghana, 2017–2019*

Isolate	ENA sample accession no.	Readfile†	No. reads	No. bases	Average read length after trimming, nt	Isolate estimated genomic coverage depth
KP040	ERS4413702	Forward	722,139	170,235,297	235.7	61.9
KP040	ERS4413702	Reverse	722,139	170,426,908	236.0	61.9
KP0033	ERS4413703	Forward	964,601	202,027,639	209.4	73.5
KP0033	ERS4413703	Reverse	964,601	202,477,205	209.9	73.5
KP0455	ERS4413704	Forward	829,523	175,627,189	211.7	63.9
KP0455	ERS4413704	Reverse	829,523	176,053,137	212.2	63.9
KP0457	ERS4413705	Forward	1,125,573	237,891,265	211.4	86.6
KP0457	ERS4413705	Reverse	1,125,573	238,338,411	211.7	86.6
KP0879	ERS4413706	Forward	1,061,700	224,561,488	211.5	81.7
KP0879	ERS4413706	Reverse	1,061,700	225,022,459	211.9	81.7
KP2201	ERS4413707	Forward	744,282	179,536,276	241.2	65.3
KP2201	ERS4413707	Reverse	744,282	179,682,159	241.4	65.3
KP2326	ERS4413708	Forward	698,537	161,304,000	230.9	58.7
KP2326	ERS4413708	Reverse	698,537	161,608,087	231.4	58.7
KP2455	ERS4413709	Forward	991,694	228,665,771	230.6	83.2
KP2455	ERS4413709	Reverse	991,694	229,174,707	231.1	83.2
KP2557	ERS4413710	Forward	582,358	139,224,749	239.1	50.7
KP2557	ERS4413710	Reverse	582,358	139,386,695	239.3	50.7
KP007	ERS4413711	Forward	1,312,323	250,115,269	190.6	91.1
KP007	ERS4413711	Reverse	1,312,323	250,983,471	191.3	91.1
KP010	ERS4413712	Forward	1,065,476	200,355,942	188.0	73.0
KP010	ERS4413712	Reverse	1,065,476	201,146,156	188.8	73.0
KP011	ERS4413713	Forward	797,568	163,213,177	204.6	59.5
KP011	ERS4413713	Reverse	797,568	163,895,685	205.5	59.5
KP025	ERS4413714	Forward	760,697	155,069,123	203.9	56.5
KP025	ERS4413714	Reverse	760,697	155,927,048	205.0	56.5
KP034	ERS4413715	Forward	738,243	151,437,269	205.1	55.2
KP034	ERS4413715	Reverse	738,243	152,116,537	206.1	55.2
KP035	ERS4413716	Forward	841,530	168,652,797	200.4	61.5
KP035	ERS4413716	Reverse	841,530	169,680,966	201.6	61.5
KP036	ERS4413717	Forward	619,995	123,825,759	199.7	45.1
KP036	ERS4413717	Reverse	619,995	124,404,982	200.7	45.1
KP037	ERS4413718	Forward	565,982	108,721,119	192.1	39.7
KP037	ERS4413718	Reverse	565,982	109,364,092	193.2	39.7
KP045	ERS4413719	Forward	599,990	111,415,949	185.7	40.6
KP045	ERS4413719	Reverse	599,990	112,065,980	186.8	40.6
KP047	ERS4413720	Forward	661,622	139,851,520	211.4	50.9
KP047	ERS4413720	Reverse	661,622	140,193,869	211.9	50.9
KP052	ERS4413721	Forward	532,921	105,873,306	198.7	38.6
KP052	ERS4413721	Reverse	532,921	106,199,103	199.3	38.6
KP055	ERS4413722	Forward	503,906	99,810,777	198.1	36.5
KP055	ERS4413722	Reverse	503,906	101,047,633	200.5	36.5
KP056	ERS4413723	Forward	763,767	149,354,936	195.6	54.6
KP056	ERS4413723	Reverse	763,767	150,709,998	197.3	54.6
KP058	ERS4413724	Forward	795,791	157,088,400	197.4	57.3
KP058	ERS4413724	Reverse	795,791	158,317,870	198.9	57.3
KP221	ERS4413725	Forward	975,198	190,938,388	195.8	69.7
KP221	ERS4413725	Reverse	975,198	192,680,609	197.6	69.7
KP233	ERS4413726	Forward	982,201	191,126,716	194.6	69.7
KP233	ERS4413726	Reverse	982,201	192,409,941	195.9	69.7
KP242	ERS4413727	Forward	827,894	157,712,667	190.5	57.6
KP242	ERS4413727	Reverse	827,894	159,032,043	192.1	57.6
KP026	ERS4413728	Forward	1,217,633	262,764,905	215.8	95.8
KP026	ERS4413728	Reverse	1,217,633	264,143,628	216.9	95.8
KP090	ERS4413729	Forward	980,113	208,425,456	212.7	76.0
KP090	ERS4413729	Reverse	980,113	209,594,572	213.8	76.0

*ENA, European Nucleotide Archive.
†Forward 5′→3′ read direction, Reverse 3′→5′ read direction.

Appendix Table 2. Time-based phylogenetic analyses of *Klebsiella pneumoniae* isolates from a neonatal intensive care unit, Ghana, 2017–2019*

Outgroup used	tMRCA (mean)	tMRCA (median)	95% Highest posterior density interval	Minimum effective sample size value for estimated parameters	Estimated mean substitution rate (SNPs/site/year)	Estimated mean substitution rate (SNPs/year)
None	2017.1	2017.1	2016.6–2017.5	4427	2.1×10^{-6}	10.0
KP055	2017.2	2017.3	2016.6–2017.6	1838	2.2×10^{-6}	10.4
Reference strain HS11286 included and as outgroup	2017.3	2017.3	2017.0–2017.6	2684	2.1×10^{-6}	9.9

*SNP, single-nucleotide polymorphism; tMRCA, time of most recent common ancestor.

Appendix Table 3. Possible combinations of the initially tested models and priors of *Klebsiella pneumoniae* isolates from a neonatal intensive care unit, Ghana, 2017–2019*

Variation of time-based phylogenetic analysis	Minimum effective sample size value for estimated parameters	Maximum effective sample size value for estimated parameters	tMRCA, mean	95% Highest posterior density interval upper bound	95% Highest posterior density interval lower bound
GTR + relaxed log normal clock + coalescent constant population	4,790	43,623	2017.07	2017.49	2016.57
GTR + relaxed exponential clock + coalescent exponential population	3,015	75,826	2017.05	2017.61	2016.17
GTR + relaxed log normal clock + coalescent exponential population	3,448	45,087	2017.06	2017.48	2016.56
HKY + relaxed log normal clock + coalescent exponential population	4,553	52,925	2017.06	2017.48	2016.56
HKY + relaxed exponential clock + coalescent exponential population	3,037	77,860	2017.05	2017.61	2016.17
HKY + relaxed log normal clock + coalescent constant population	2,762	44,916	2017.07	2017.49	2016.57
HKY + relaxed exponential clock + coalescent constant population	4,175	77,924	2017.08	2017.61	2016.26
GTR + strict clock + coalescent exponential population	3,015	75,826	2017.07	2017.42	2016.67
HKY + strict clock + coalescent constant population	5,822	96,513	2017.08	2017.43	2016.68
GTR + strict clock + coalescent constant population	4,808	94,467	2017.08	2017.42	2016.68
HKY + strict clock + coalescent exponential population	4,537	99,710	2017.07	2017.42	2016.67
GTR + relaxed exponential clock + coalescent constant population	4,677	83,831	2017.08	2017.61	2016.26

*GTR, general time-reversible; HKY, Hasegawa, Kishino, and Yano 1985; tMRCA, time of most recent common ancestor.

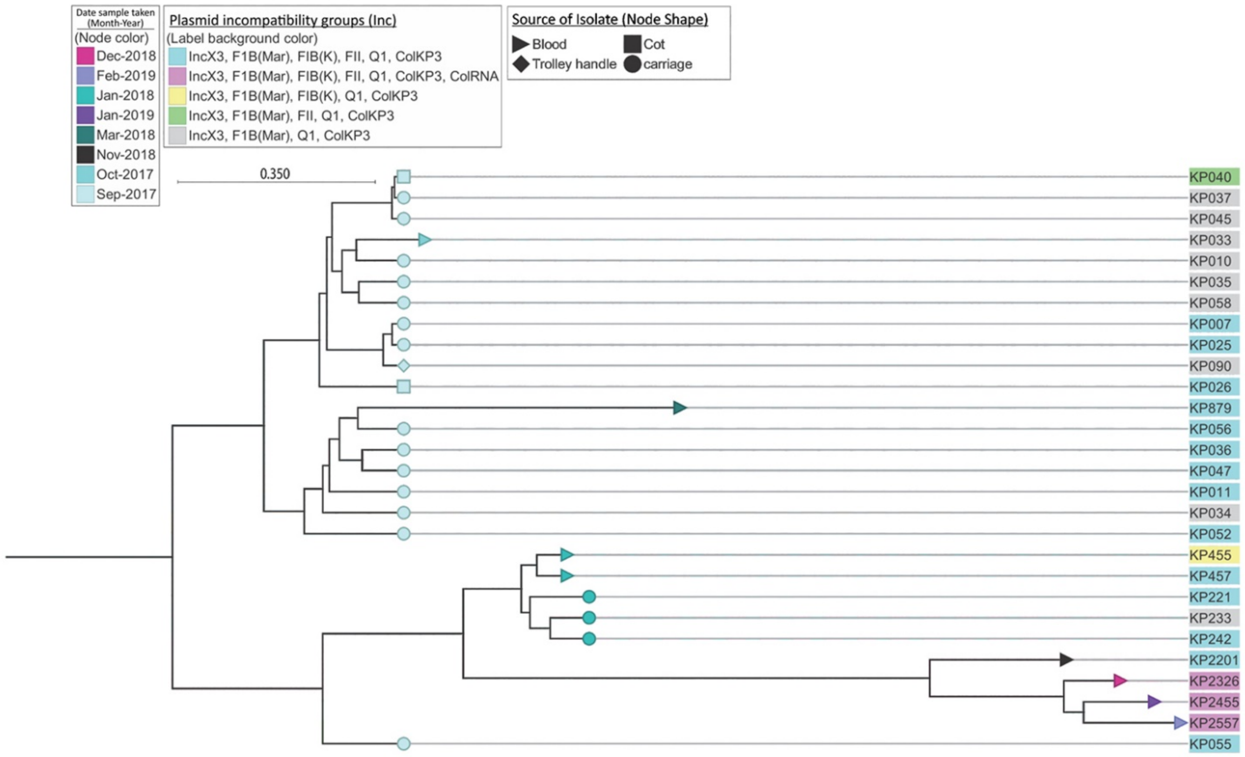

Appendix Figure. Time-based phylogenetic tree of *Klebsiella pneumoniae* isolates from the neonatal intensive care unit at Korle-Bu Teaching Hospital, Accra, Ghana, 2017–2019. Genome of *K. pneumoniae* reference strain HS11286 used as outgroup. Time of most recent common ancestor of the outbreak estimated as April 2017 (year 2017.3; 95% highest posterior density interval 2017.0–2017.6) with an estimated mean substitution rate of 2.1 × 10^{-6} single-nucleotide polymorphisms/site/year (9.9 polymorphisms/year).
